# The First Vietnamese Patient of LEOPARD Syndrome due to a *PTPN11* Mutation: A Case Report and Review of the Literature

**DOI:** 10.1155/2021/8197435

**Published:** 2021-09-13

**Authors:** Hao Trong Nguyen, Nguyen Nhat Pham, Hoang Anh Vu, Tu Nguyen Anh Tran

**Affiliations:** ^1^Ho Chi Minh City Hospital of Dermato-Venereology, Ho Chi Minh City, Vietnam; ^2^Center for Molecular Biomedicine, University of Medicine and Pharmacy at Ho Chi Minh City, Ho Chi Minh City, Vietnam

## Abstract

LEOPARD syndrome is a rare congenital anomaly that involves several organs. Patients with this syndrome develop multiple lentigines resembling a leopard's hide. LEOPARD is an acronym of the major features constituting the syndrome including lentigines, electrocardiographic conduction defects, ocular hypertelorism, pulmonary valve stenosis, anomalies of genitalia, retardation of growth, and deafness. The syndrome is rare, and only 200 cases have been reported yet worldwide. We present the case of an 8-year-old female patient who visited the Ho Chi Minh City Hospital of Dermato-Venereology because of multiple brownish-black “dots” on her face and body. On examination, she also showed abnormalities in the maxillofacial bones, vertebrae, shoulders, sternum, and teeth, as well as deaf-mutism and growth retardation, which are typical of LEOPARD syndrome. Genetic analysis revealed a *PTPN11* gene mutation in this case. To the best of our knowledge, this is the first case of LEOPARD syndrome reported in Vietnam.

## 1. Introduction

Zeiler and Becker reported distinct features of LEOPARD syndrome for the first time in 1936 [1]. Over two decades later, in 1969, Gorlin et al. aggregated the different components into one syndrome and coined the term “LEOPARD syndrome.” LEOPARD is an acronym of the components of the syndrome and includes lentigines, electrocardiographic conduction defects, ocular hypertelorism, pulmonary valve stenosis, abnormalities of genitalia, retardation of growth, and deafness. The syndrome is a congenital defect caused by the mutation of *PTPN11* (90%), *RAF1*, or *BRAF* genes and is so rare that only approximately 200 cases have been reported worldwide [2, 3]. No cases of this syndrome have yet been reported in Vietnam.

## 2. Case Presentation

**Figure 1:**
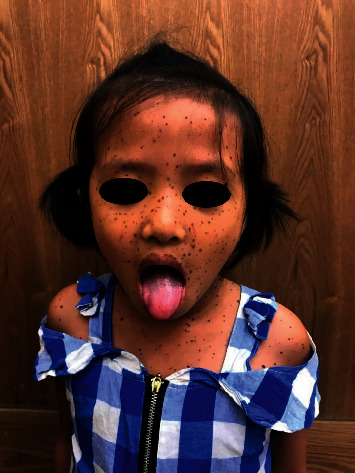
The picture of the presented 8-year-old patient. *Note.* Multiple brownish-black “dots” are discretely distributed on the patient's face and upper trunk.

**Figure 2:**
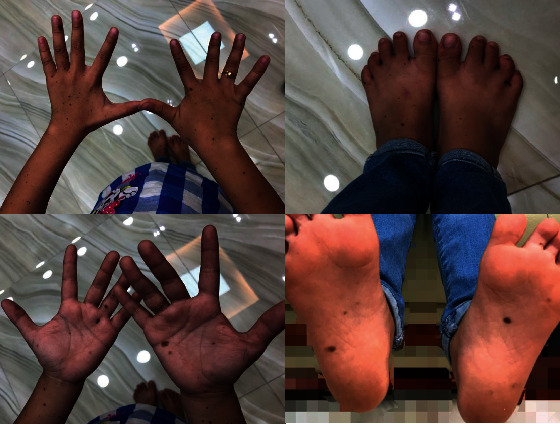
Lentigines distribution on the patient's extremities.

**Figure 3:**
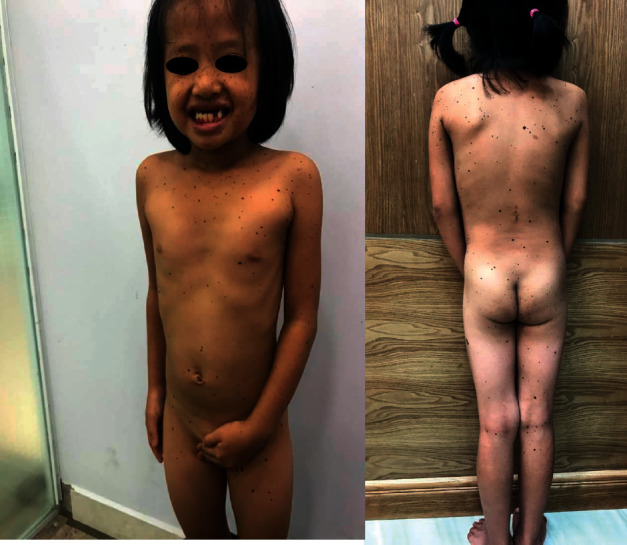
The distribution of the lentigines on the patient's trunk on further physical examination.

**Figure 4:**
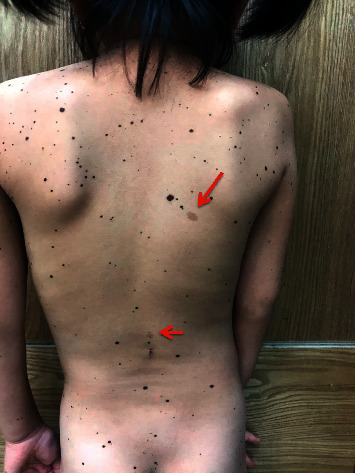
Café-au-lait macules along with lentigines. *Note.* Scoliosis and protruding shoulder blade from the back (scapula alata) can be seen easily from this aspect.

**Figure 5:**
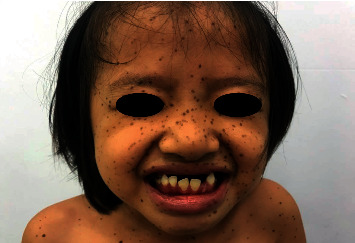
The facial characteristics of the patient. *Note.* Along with lentigines, a flat nasal bridge, hypertelorism, and abnormal dental development are present.

**Figure 6:**
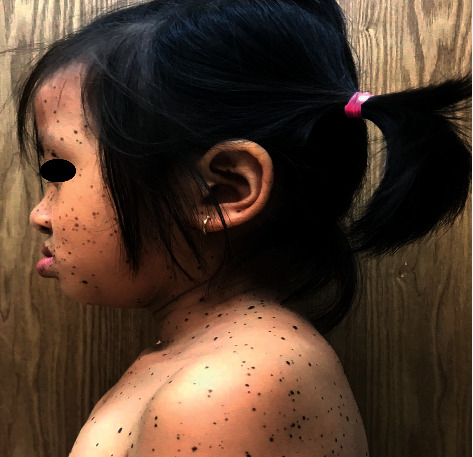
The lateral aspect shows a protruding lower jaw (prognathism), a flat nasal bridge, and low-set ear. A part of the protruding chest (pectus carinatum) is also seen.

An 8-year-old female patient visited Ho Chi Minh City Hospital of Dermato-Venereology because of several brownish-black “dots” on her face and body (Figure 1). This condition began to appear at her fourth year of age, and the quantity of the macules increased with age. She also presented with congenital deaf-mutism and retarded growth. No recognized history of family members with a similar condition was noted. Physical examination showed multiple brownish-black macules, varying in shape and size, and with diameters ranging from 1 mm to 5 mm. The macules were discretely spread across the child's face, chest, back, upper and lower extremities, and palms and soles (Figures 2 and 3). Café-au-lait macules of size 2 × 3 cm^2^ were observed on the back (Figure 4). Distinct facial features included hypertelorism, a flat nasal bridge, and prognathism (protruding lower jaw) (Figures 5 and 6). Dental arch discrepancies, diastema, and hypodontia were also noted (Figure 6). Pectus carinatum (protruding chest) (Figures 2 and 6), scoliosis, and scapula alata (Figure 4) were observed. The patient showed growth retardation with a height and weight of 105 cm and 15 kg, respectively.

**Figure 7:**
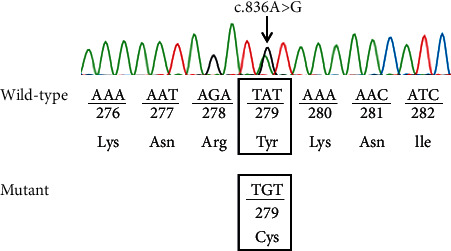
Genetic analysis of the PTPN11 gene. The analysis was performed using the ABI 3500 genetic analyzer system. The results show a heterozygous mutation on exon 7 of the aforementioned gene.

The radiologic and laboratory workups revealed certain notable findings. The dental roots were abnormal or absent in some positions, as seen on the radiograph. Uterine hypoplasia was noted on abdominal ultrasound and was comparable with similar findings among those of the same age. Genetic analysis revealed a *PTPN11* gene mutation (c.836A > *G*, p.Tyr279Cys) (Figure 7). No abnormalities were seen on electrocardiogram (ECG), echocardiogram, and chest radiograph (CXR).

On collating and considering these signs and symptoms, we concluded that the most likely diagnosis of the patient's condition was LEOPARD syndrome.

## 3. Discussion

Lentigines are the most prominent feature of LEOPARD syndrome. They range approximately 3–5 mm in diameter and often present on the skin of the neck, upper and lower extremities, and trunk. Lentigines can also be seen on the face, scalp, palms, soles, and genital area; however, no case had been reported with mucous membrane involvement. The lentigines begin to appear at the age of 4-5 years and spread over time without any relationship with sunlight exposure [1]. In addition to lentigines, approximately 50% of the patients have café-au-lait macules [4].

Besides the manifestations on the skin, other organs of patients may also be involved in syndrome. A flat nasal bridge, low-set ears, or defects in the shapes of ears can be observed in 87% of patients with LEOPARD syndrome [1]. Almost all patients have hypertelorism, which is an abnormal increase in the distance between the two eye orbits. Patients may also have congenital ptosis, prominent lips, and macroglossia.

Notably, 70–85% of the patients with LEOPARD syndrome have cardiovascular involvement, such as hypertrophic cardiomyopathy (70%), pulmonary valve stenosis (25%), or more rarely, aortic and bicuspid valve malformations [4]. Hypertrophic cardiomyopathy is the most frequently seen cardiovascular involvement that may be fatal. Additionally, 75% of the patients have electrocardiographic changes including P-waves abnormalities (19%), prolonged QT-interval (23%), conduction abnormalities (23%), and impaired repolarization (42%) [1].

Deformities of the skeletal and genitourinary systems are usually observed in patients with LEOPARD syndrome. Approximately 75% have chest deformities, prognathism, scapula alata (protruding shoulder blade from the back), scoliosis (lateral curvation of the spine), or ankyloses of the spine. Chest deformations may also present as pectus carinatum (protruding chest) or pectus excavatum (sunken chest) [1]. Nearly 50% of the male patients also have bilateral cryptorchidism, hypospadias, or hypogonadism. Besides, the female patients often have delayed puberty and ovarian hypoplasia. Additionally, horseshoe kidneys can also manifest in this syndrome.

Furthermore, approximately 85% of the patients experience growth and/or mental retardation to a varying extent, and sensorineural hearing loss can present in 15–25% of the cases [1].

The diagnosis of LEOPARD syndrome largely depends on the clinical examination. In 1976, Voron et al. proposed criteria for the syndrome. Based on the criteria, a patient was diagnosed with the syndrome if they had multiple lentigines on their body and showed more than two of the components constituting the LEOPARD acronym. However, three other components of the acronym needed to be present to diagnose the syndrome if the patient did not have lentigines [1].

Apart from the multiple lentigines that appeared when she was 4 years old, our patient also presented with café-au-lait macules on the back and four of the six other components of LEOPARD syndrome including ocular hypertelorism, abnormal genitalia, retardation of growth, and sensorineural deafness. Thus, she fulfilled the diagnostic criteria for LEOPARD syndrome. For more objective evidence, a genetic analysis was performed for the patient in the Center for Molecular Biomedicine, University of Medicine and Pharmacy at Ho Chi Minh City. The result showed that she had *PTPN11* gene mutation which is appropriate to previous literature; LEOPARD syndrome results from the mutation of *PTPN11*, *RAF1*, or *BRAF* genes, and 90% of the reported cases had a mutation in the *PTPN11* gene [3, 5].

To the best of our knowledge, the latest case worldwide was reported in 2017 by Cançado et al. [3]. The case was of a 12-year-old male Brazilian patient with multiple lentigines, ocular hypertelorism, macroglossia, dental defects, hypospadias, cryptorchidism, aortic valve stenosis, growth retardation, and 50% hearing loss. His ECG also showed prolonged PR intervals.

Previously, in 2015, there is a case of a 22-year-old woman with the syndrome reported in India [4]. Aside from multiple lentigines and café-au-lait macules of the face, neck, trunk, and extremities, this patient also presented with ocular hypertelorism, flat nasal bridge, deafness, growth, mental retardation, polycystic ovary syndrome on abdominal ultrasound, right ventricular hypertrophy on echocardiogram, and prolonged QT-intervals on ECG.

In literature, the prognosis of LEOPARD syndrome varies by the extent of the involvements of the cardiovascular and other systems. Fortunately, the presented patient had a more favorable prognosis than previously reported cases since she did not have such extensive system involvements. However, reports show that in 70–85% of the patients who have cardiovascular abnormalities, the abnormalities may manifest congenitally but often become detectable after the appearance of lentigines [4]. These abnormalities may gradually progress with the spread of the lentigines. Therefore, ECG, CXR, and echocardiography should be performed annually for our patient.

Urs et al. recognized an interesting clinical characteristic, namely, the dental defects, in their patient and previously reported cases [6]. They presented the case of a 4-year-old male patient with the chief complaint of dental caries. Physical examination revealed discrete multiple lentigines on the face, trunk, and hands. Ocular hypertelorism, flat nasal bridge, and thick lips were also remarkable. The dental examination showed diastema, hypodontia in some positions, and other anomalies. The patient also experienced hypothyroidism and asymmetric septal hyperplasia. It was hypothesized that the LEOPARD syndrome resulted from the abnormalities in the neural crest development which in turn led to the abnormalities in the organs that originated from the neural crest. These organs include melanocytes, a part of the central nervous system, craniofacial bones, auditory vesicles, and tooth buds. With this hypothesis and the patient's chief complaint of dental caries and development, Urs et al. recommended that dental abnormalities could be early signs that suggest the diagnosis of LEOPARD syndrome when the skin manifestations and organ involvement were not obvious. They also proposed that dental and craniofacial anomalies may be added to the *D* component of the LEOPARD acronym.

As seen in our patient, the three aforementioned cases and other reported cases did not have a positive family history of the syndrome. However, in 2014, there was a solitary report that five people in a 2-generation family had LEOPARD syndrome in Bosnia and Herzegovina. Genetic analysis revealed that all of five people had *PTPN11* gene mutation although their clinical manifestations differed from each other [7].

To the best of our knowledge, we report the first case of LEOPARD syndrome in Vietnam. In addition to the abnormal pigmentation of the skin and congenital anomalies of other organs, genetic abnormality should be considered in such cases, and the genetic analysis would be appropriate to confirm the diagnosis.

## Data Availability

The laboratory test and clinical data used to support the findings of this study are included within the article.
